# Characterization of the interactions between the active site of a protein tyrosine kinase and a divalent metal activator

**DOI:** 10.1186/1471-2091-6-25

**Published:** 2005-11-23

**Authors:** Xiaofeng Lin, Marina K Ayrapetov, Gongqin Sun

**Affiliations:** 1 Department of Cell and Molecular Biology, University of Rhode Island, Kingston, RI 02881, USA

## Abstract

**Background:**

Protein tyrosine kinases are important enzymes for cell signalling and key targets for anticancer drug discovery. The catalytic mechanisms of protein tyrosine kinase-catalysed phosphorylation are not fully understood. Protein tyrosine kinase Csk requires two Mg^2+ ^cations for activity: one (M1) binds to ATP, and the other (M2) acts as an essential activator.

**Results:**

Experiments in this communication characterize the interaction between M2 and Csk. Csk activity is sensitive to pH in the range of 6 to 7. Kinetic characterization indicates that the sensitivity is not due to altered substrate binding, but caused by the sensitivity of M2 binding to pH. Several residues in the active site with potential of binding M2 are mutated and the effect on metal activation studied. An active mutant of Asn319 is generated, and this mutation does not alter the metal binding characteristics. Mutations of Glu236 or Asp332 abolish the kinase activity, precluding a positive or negative conclusion on their role in M2 coordination. Finally, the ability of divalent metal cations to activate Csk correlates to a combination of ionic radius and the coordination number.

**Conclusion:**

These studies demonstrate that M2 binding to Csk is sensitive to pH, which is mainly responsible for Csk activity change in the acidic arm of the pH response curve. They also demonstrate critical differences in the metal activator coordination sphere in protein tyrosine kinase Csk and a protein Ser/Thr kinase, the cAMP-dependent protein kinase. They shed light on the physical interactions between a protein tyrosine kinase and a divalent metal activator.

## Background

Protein tyrosine kinases (PTK)^1 ^are a large family of enzymes that transfer the γ-phosphate of ATP to tyrosine hydroxyl groups in proteins. By phosphorylation, PTKs regulate the conformation and function of their protein substrates [1]. This covalent modification is a fundamental mechanism of signal transduction in mammalian cells. Aberrant activation of many specific protein tyrosine kinases causes mishaps in cell signalling, and results in proliferative diseases, such as cancer [2]. Many protein tyrosine kinases are considered as important targets for drug development against such diseases [3]. For full understanding of phosphorylation-mediated signalling and to provide a knowledge base for anti-PTK drug discovery, it is important to understand the catalytic mechanisms of protein tyrosine kinases.

C-terminal Src kinase (Csk) is a cytoplasmic PTK that phosphorylates Src family kinases (SFKs) and down-regulates their kinase activities [4,5]. The mechanistic basis of catalysis by Csk and PTKs in general is still poorly understood. Csk-catalyzed phosphorylation reaction obeys a ternary complex mechanism, likely with rapid and random binding of ATP-Mg and the phosphate-accepting substrate [6]. In addition to a Mg^2+ ^cation (M1) as part of the ATP-Mg complex, Csk requires another Mg^2+ ^ion (M2) for optimal kinase activity [7,8]. Kinetic studies demonstrate that M2 is an essential activator [7]. Because the affinity of Csk for the metal activator at 2.3 mM falls within the range of the cellular Mg^2+ ^concentration, this activation may play a regulatory role in the kinase function [7,9].

Even though Mg^2+ ^is likely the physiological activator, several other divalent metal cations can substitute for Mg^2+ ^and activate Csk to various levels [8,10]. For example, Mn^2+ ^can replace Mg^2+ ^and results in higher activity of Csk, while Co^2+^, Ni^2+ ^are not as effective as Mg^2+ ^as an activator. Zn^2+ ^can also substitute for Mg^2+ ^in binding to the M2 binding site, but it cannot serve as an activator. Thus, Zn^2+ ^acts as an inhibitor of Csk activity competitive against M2 [8]. Another intriguing property of the Csk-metal interaction is that these substitution metals all bind to Csk considerably stronger than the physiological activator, Mg^2+^. While Csk binds to Mg^2+ ^with an AC_50 _of 2.3 mM, the other metal cations all bind to Csk with AC_50 _or IC_50 _in the low μM range. Among all divalent metal cations tested, Zn^2+ ^has the highest affinity for Csk, with an IC_50 _of 0.5 μM [8].

The requirement of two divalent metal cations for full activity by Csk appears to represent a general catalytic requirement by all PTKs. Several PTKs from different families, such as v-Fps [11], Yes [12], Src [13], Lck [14], insulin receptor kinase [15] and epidermal growth factor receptor [16], all require two Mg^2+ ^cations for full activity. The insulin receptor kinase has been co-crystallized with both a peptide substrate and an ATP analog [17]. In the active site, two Mg^2+ ^are observed, providing direct structural evidence for the presence of two Mg^2+ ^ions in PTK catalysis. Kinetic analysis reveals that the metal cation activator might participate in catalysis by different mechanisms for different PTKs. For example, M2 activates Csk and Src by increasing the k_cat _without affecting the K_m _for ATP [7]. However, M2 activates IRK [18] and v-Fps [11] by decreasing the K_m _for ATP without affecting the k_cat_. The mechanistic basis for such kinetic differences has not been determined.

Interestingly, a protein Ser/Thr kinase, the cAMP-dependent protein kinase (PKA), also binds to two divalent metal cations in the active site during catalysis [19]. However, the second Mg^2+ ^inhibits the kinase activity [20]. Crystallization of PKA complexed with catalytic ligands reveals that two Mg^2+ ^cations are present in the active site [21].

In the current study, we characterized the parameters for the interactions between Csk and M2, such as activity sensitivity to pH, required physical parameters of the divalent metal cation activators, and potential M2 coordinating residues. Mutagenic studies eliminated a residue as a potential ligand for M2, but could not determine if two other residues are involved due to lack of activity in all mutants varying these residues.

## Results

### Csk activity is sensitive to pH in the range of 6 to 7

**Figure 1:**
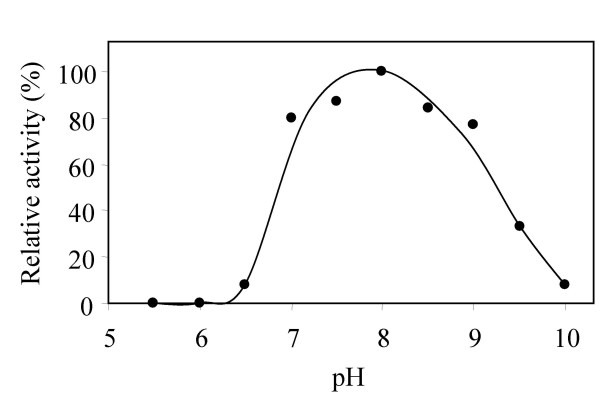
**pH optimum of Csk**. The Csk kinase activity is assayed as described in the Methods section. The activity at different pH is normalized to that at pH 8.

Because many PTKs are molecular targets for drug discovery, it is of high interest to understand the mechanisms of PTK catalysis. Like most protein tyrosine kinases, the catalytic mechanism of Csk is not fully understood. Csk has a bell-shaped pH response curve, with a pH optimum of around 8 (Figure 1). In the acidic arm of the pH curve, the activity is highly sensitive to pH. At pH 6, the enzyme shows very little activity, but at pH 7, the enzyme is nearly fully active. We hypothesize that this sensitivity likely reflects certain catalytically essential step(s) that is carried out by a functional group with an apparent pK_a _in this range. Elucidation of such catalytically essential step(s) may shed light on the catalytic mechanism.

**Figure 2:**
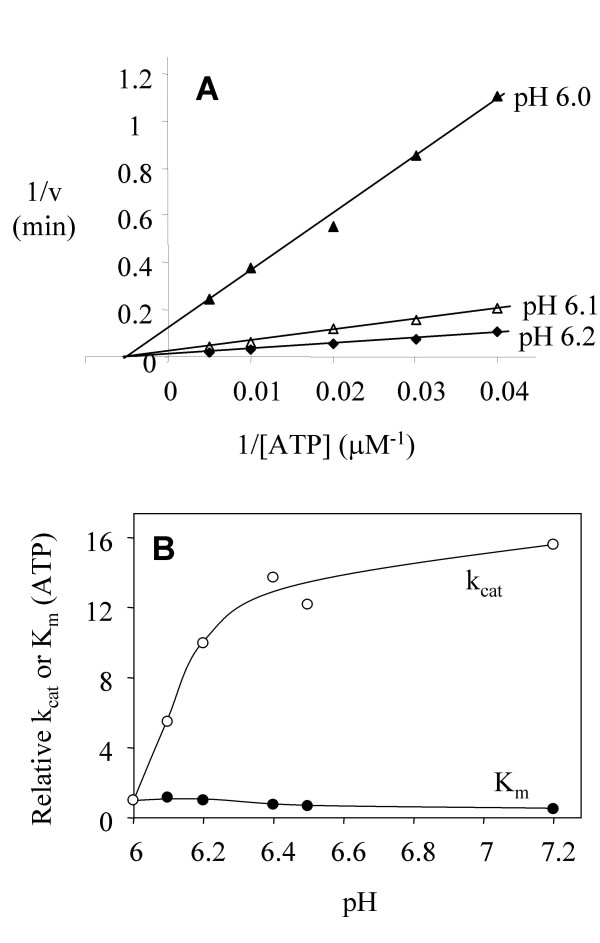
**Effect of pH on Csk kinetics with ATP as the variable substrate**. (A) Double reciprocal plot of Csk activity using ATP as the variable substrate at pH 6, 6.1 and 6.2. (B) Plots of relative k_cat _and K_m _for ATP as a function of pH. The phosphate-accepting substrate was polyE_4_Y at 1 mg ml^-1^. The k_cat _(9.1 min^-1^) and K_m _(200 μM) at pH 6 are taken as 1 and all other values are relative to those.

To test the above hypothesis, we characterized how steady state catalytic parameters responded to pH changes in this range (Figure 2). We first determined the catalytic parameters using ATP as the variable substrate. For this purpose, polyE_4_Y, a random polymer of Glu and Tyr (4:1), was used as the phosphate-accepting substrate at a fixed concentration of 1 mg ml^-1^. The apparent K_m _for ATP decreased from 200 μM at pH 6 to 91 μM at pH 7.2 (relative K_m _from 1 to 0.45 in Figure 2), but the k_cat _increased approximately 16 fold. The pK_a _for this k_cat _change is estimated to be 6.2. This indicates that phosphoryl transfer but not ATP binding to Csk is sensitive to pH. This pattern is different from those for PKA, whose apparent K_m _for ATP gradually decreases by a factor of 7 when pH increases from 5.5 to 7 [22]. This contrast in kinetic patterns likely reflects differences in participation by pH-sensitive functional groups in catalysis by these two enzymes. In PKA, the sensitivity to pH is due to increased binding of ATP-M1 at a higher pH. Asp184 is a coordination ligand for M1 [21]. For Csk, the apparent K_m _for ATP is also dependent on the presence of M1, with M1 resulting in lower K_m _for ATP [7]. However, because the apparent K_m _of Csk for ATP was largely independent of pH, the function of M1 is likely not affected by pH in this range. The contrast between Csk and PKA suggests that M1 coordination in Csk and PKA is different.

**Figure 3:**
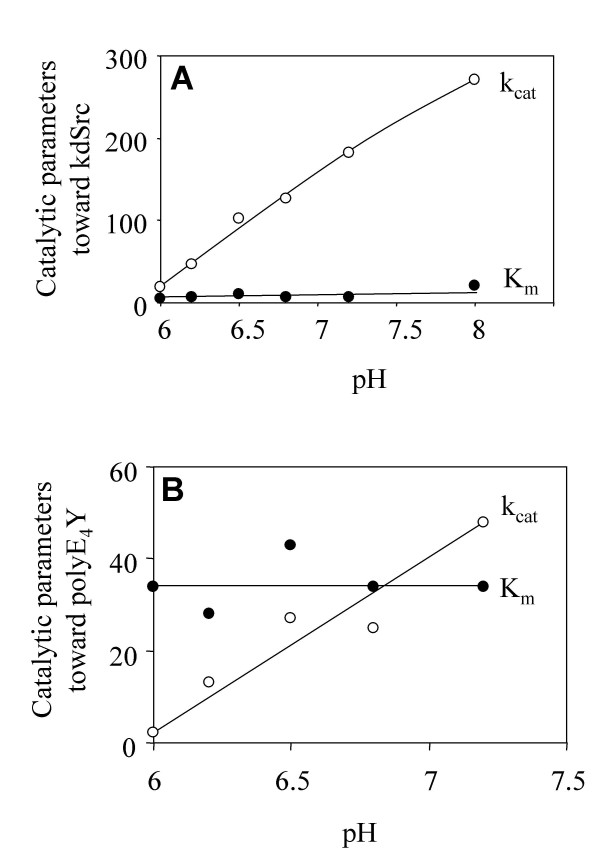
**Effect of pH on Csk catalytic parameters using phosphate-accepting substrate as the variable substrate**. (A) KdSrc is used as the variable substrate. (B) PolyE_4_Y is used as the variable substrate.

Steady state kinetics was then performed using the phosphate-accepting substrate as the variable substrate. The physiological substrates for Csk are the Src family kinases. Because SFKs are themselves PTKs that autophosphorylate, we used a kinase-defective mutant of Src (kdSrc) as the substrate. KdSrc contains a point mutation in the active site (Lys295Met), which abolishes Src kinase activity but does not affect its ability to serve as a Csk substrate [23,24]. The K_m _of Csk for kdSrc did not change significantly in response to pH in the range of 6 to 7. However, the k_cat _increased over 100-fold (Figure 3A). A similar kinetic pattern was observed for polyE_4_Y, a commonly used artificial substrate for PTK activity assays (Figure 3B) [7,8]. This result indicates that the recognition of the phosphate-accepting substrate is not affected by pH changes in this range. Because the k_cat _may be a function of both the phosphoryl transfer step and the ADP release [25], one of these steps is likely affected by pH in this range.

### M2 binding to Csk is sensitive to pH

**Figure 4:**
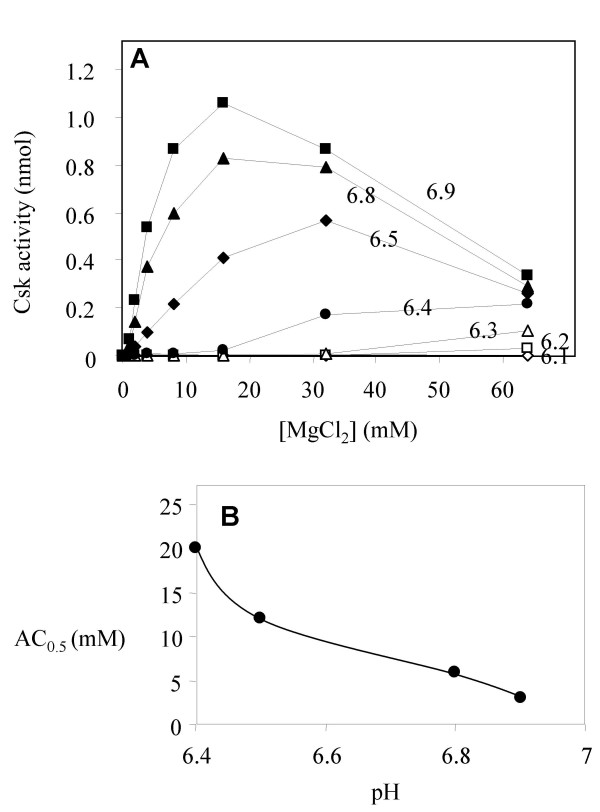
**Mg^2+ ^activation of Csk as a function of pH**. (A) Mg^2+ ^activation of Csk is determined at different pH. The pH values are labeled in the graph. (B) Plot of AC_50 _as a function of pH.

Because phosphoryl transfer, not substrate binding is sensitive to pH, it is likely that certain functional groups that catalyze phosphoryl transfer are subject to ionization in this pH range. Two types of functional groups could fall within this category. A functional group may directly participate in catalysis by interacting with the transition state, or it may interact with an essential activator. If only one ionization state of such a group is functional, it would render Csk phosphoryl transfer sensitive to pH. Because M2 acts as an essential activator, we determined if M2 binding to Csk was sensitive to pH in this range. The affinity of Csk for M2 can be roughly measured by the AC_50_, the concentration of Mg^2+ ^that activates Csk to 50% of its full activity [7]. For example, at the optimal pH (8.0), Mg^2+ ^activates Csk with an AC_50 _of 2.3 mM. We determined if the AC_50 _of Csk for Mg^2+ ^activation was sensitive to pH. As shown in Figure 4A, the ability of Mg^2+ ^to activate Csk was indeed sensitive to pH. At pH 6.9, the AC_50 _was 3 mM, close to the optimal AC_50_. As pH decreased, progressively higher concentration of Mg^2+ ^was required for Csk activation. At pH 6.4, 64 mM MgCl_2 _nearly saturated Csk, and the AC_50 _was estimated to be 20 mM. At pH 6.3 or below, highest activity was detected at 64 mM, making it difficult to estimate an AC_50_, but it is clear AC_50 _continued to increase as pH decreased. It is known that Csk activity is highly sensitive to ionic inhibition [26], making it difficult to separate the effect of Mg^2+ ^as an activator from ionic inhibition at high MgCl_2 _concentrations. Despite the lack of an accurate determination of the relationship between AC_50 _and pH, it is clear that the AC_50 _of Csk for Mg^2+ ^is dependent on pH. The pH dependence of metal binding is further illustrated by the pH dependence profile in Figure 4B. The pK_a _of this function was estimated in the range of 6.2 to 6.5, which correlated to the pH range where Csk activity was most sensitive to pH. This result strongly suggests that the sensitivity of phosphoryl transfer to pH is at least partly due to sensitivity of M2 binding to pH. Because the activity is gained with increased pH, the deprotonated form of the functional group is responsible for M2 binding.

### Characterization of potential metal-coordinating residues in the active site of Csk

Only one protein tyrosine kinase, the insulin receptor kinase, has been co-crystallized with substrate analogs and divalent metal activators [17]. We compared the structures of Csk and IRK to identify Csk residues potentially involved in M2 coordination. In IRK, three residues are involved in metal cation coordination, Asp1150, Glu1047, and Asn1137. All three residues are conserved among PTKs, corresponding to Asp332, Glu236 and Asn319 in Csk [27]. Even though Csk and IRK displayed some differences in the kinetic patterns of Mg^2+ ^activation, it is likely that the conserved residues are playing similar roles in Mg^2+ ^coordination. We performed site-specific mutagenesis on these residues to determine if they are involved in metal activator coordination in Csk.

**Figure 5:**
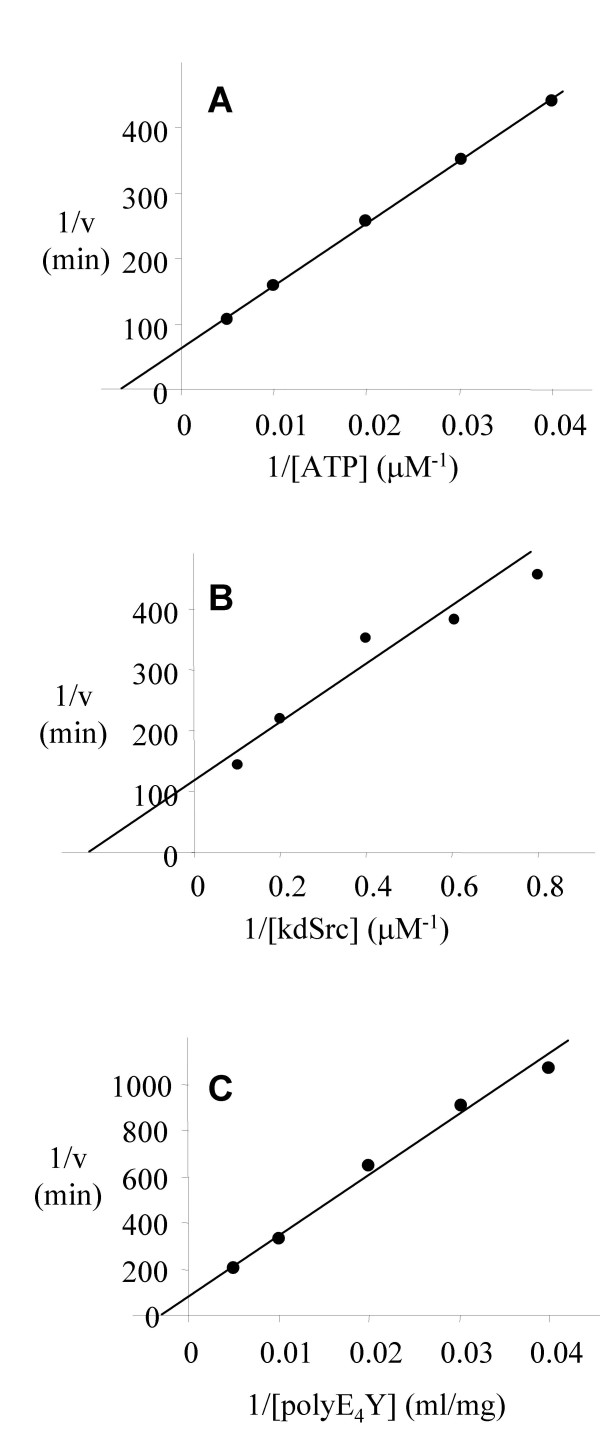
**Kinetic analysis of Asn319Ser**. Double reciprocal plots of Asn319Ser, using ATP (A), kdSrc (B) or polyE_4_Y (C) as the variable substrate. For the determination in (A), polyE_4_Y is used as the phosphate-accepting substrate.

**Figure 6:**
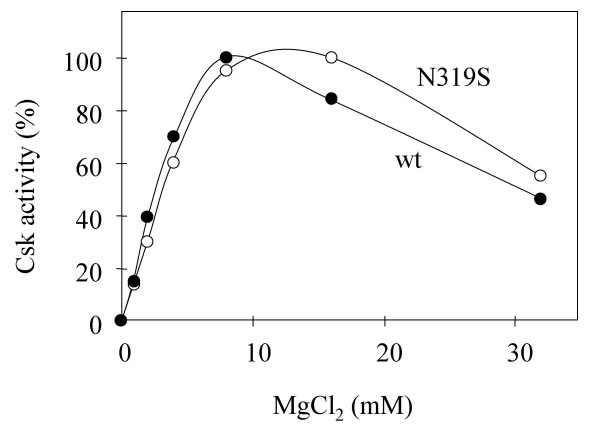
**Activation of wt and Asn319Ser mutant of Csk by Mg^2+^**. The maximum activities for both wt and Asn319Ser are set as 100%.

Asn319 is located in the catalytic loop and a universally conserved residue in all protein kinases, including Ser/Thr kinases. In IRK, the equivalent residue is involved in coordinating M1. Asn319 was mutated to Asp, His, Gln and Ser. Only one mutant, Asn319Ser, could be expressed as an active enzyme. Representative double reciprocal plots determining catalytic parameters of Asn319Ser mutant are presented in Figure 5 and summarized in Table 1. Overall, the catalytic efficiency measured by k_cat _decreased by a factor of approximately 10,000, while the apparent K_m _values for ATP, polyE_4_Y and kdSrc did not change significantly (within a factor of 2). The Mg^2+ ^activation profile of this mutant was nearly identical to that of wt Csk (Figure 6) indicating that even though this residue is crucial for Csk catalysis, it is unlikely to be responsible for the M2 coordination. Because of the large decrease in k_cat _due to the Asn319 mutation, there is a possibility that the mutation may have changed the rate-limiting step in Csk catalysis, making a direct conclusion about the role of Asn319 in M2 binding more complicated. The identical Mg^2+ ^responses by Asn319Ser and wild type Csk argue against this possibility.

**Table 1:** Catalytic parameters of Csk and Asn319Ser mutant

Parameter^a^	Wt Csk	Asn319Ser
k_cat_-ATP (min^-1^)	160 ± 10	0.01 ± 0.007
K_m_-ATP (μM)	140 ± 12	150 ± 25
k_cat_-polyE_4_Y (min^-1^)	82 ± 12	0.01 ± 0.001
K_m_-polyE_4_Y (μg ml^-1^)	156 ± 30	220 ± 48
k_cat_-kdSrc (min^-1^)	109 ± 3	0.01 ± 0.001
K_m_-kdSrc (μM)	6.4 ± 0.1	3.4 ± 0.1

^a^All assays were performed at least three times. The standard errors were calculated from three most consistent assays.

Mutation of Glu236 and Asp332 to a number of residues, Ala, Asp, Gln for Glu236, Ala, Asn, Glu for Asp332, produced inactive mutants, thus kinetic analysis of their role in M2 binding is precluded. These two residues remain likely candidates for coordinating M2, but confirmation awaits further study by other methods.

### Divalent Metal cations of certain size bind to and activate Csk

**Figure 7:**
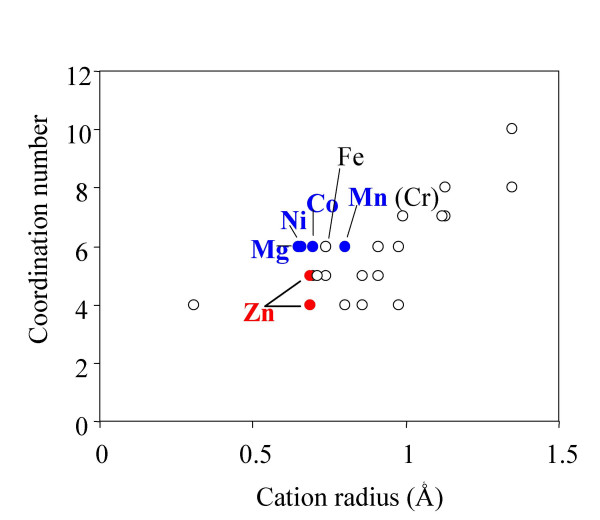
**Plot of coordination number versus cation radius for commercially available divalent metal cations**. Those cations that support Csk activity are shown in blue. Zn^2+^, which also binds to Csk but does not support Csk activity, is shown in red. Fe^2+ ^and Cr^2+ ^are also labelled. Their ability to support Csk activity is discussed in the text. The coordination numbers and the cation radii are taken from reference [28] and the inhibition data is taken from reference [8].

To characterize the physical properties required of the metal activator, it has been previously determined that Mn^2+^, Co^2+ ^and Ni^2+ ^could also serve as activators, while 14 other commercially available divalent metal cations could not [8,10]. To determine if the ability to activate Csk correlates to any specific physical attributes, we plotted the coordination number versus ionic radius [28] and determined where the activating metal cations were located on this map (Figure 7). The four divalent metal cations, Mg^2+^, Mn^2+^, Co^2+ ^and Ni^2+^, that support Csk activity clustered together with a coordination number of 6 and an ionic radius of 0.65 to 0.8 Angstroms. Two other metal cations also met these criteria but apparently did not support the kinase activity: Cr^2+ ^and Fe^2+^. Both of these metal ions have multiple valencies, and interfere with the kinase assay. Fe^2+ ^forms brown precipitates likely with the enzyme and protein substrate, while Cr^2+ ^forms precipitates with ATP in the kinase assay. Such interferences prevent a definitive analysis if they could support Csk activity. Zn^2+ ^binds to Csk tightly but does not support Csk activity, thus it inhibits Csk activity as an inhibitor competitive against M2. Zn^2+ ^also falls within the range of the ionic radius, but has a coordination number of 4 or 5. This suggests that a coordination number of 6 may be required of the metal cation activator. Although this analysis suggests that the size and coordination number are likely important factors in determining if a metal cation could activate Csk catalysis, other factors may also be important.

## Discussion

In this communication, we investigated the molecular basis of a commonly observed catalytic property of Csk. First, Csk activity is sensitive to pH change in the range of 6 to 7. Steady state kinetics demonstrates that the sensitivity is not due to the binding of Csk to either ATP-Mg or the protein substrate. The sensitivity is due to the sensitivity of M2 binding to pH in this range. Second, several residues that have the potential for M2 binding were studied by mutagenesis. These studies eliminated Asn319 in the active site as a potential ligand for M2 binding, but were inconclusive about the role of Asp332 and Glu236, because mutants at these two positions were inactive. Third, commercially available divalent metal cations were surveyed for their ability to support Csk activity. A strong correlation between the ability of divalent metal cation to support Csk activity and its physical parameters (ionic radius and the coordination number) was identified. Divalent metal cations with a coordination number of 6 and an ionic radius of 0.65–0.8 Å were able to support the activity while ions outside of this range were not. Overall this investigation provided insights into the kinase-divalent metal interaction in the active site.

The sensitivity of kinase activity to pH has been previously investigated in the cAMP-dependent protein kinase [22]. Interestingly, the binding of ATP was sensitive to pH for PKA while the binding of the second Mg^2+ ^to PKA is not sensitive to pH. This pattern is the opposite of that observed for Csk, likely reflecting different coordination patterns for M1 and M2 in Csk and PKA. This is also consistent with the structural information on IRK and PKA. Although in both PKA and IRK, three conserved residues (Glu1047, Asn1137, and Asp1150 in IRK, Glu91, Asn171 and Asp184 in PKA) are involved in coordinating M1 and M2, the positions of M1 and M2 are switched in the two kinases. In IRK, M1 is coordinated with Asn1127, while M2 is coordinated with Asp1150 directly and Glu1047 through two water molecules. In PKA, M2 is coordinated with Asn171, while M1 is coordinated with Asp184. Because M1 binding to PKA is sensitive to pH, it is likely due to deprotonation of Asp184. In this case, M2 binding to IRK would likely be sensitive to pH. This pattern is observed in Csk. This suggests that Csk and IRK likely uses a similar M2 binding site. In this case, Glu236 and Asp332 would be expected to be key ligands for M2 coordination.

Our effort to pinpoint the residues for coordinating M2 in Csk by mutagenic and kinetic studies is not fully successful. We were able to eliminate Asn319 as responsible for binding to M2, but our results are inconclusive regarding Asp332 and Glu236 due to the inability to generate active mutants at these two positions. This highlights the limitation of mutagenic approach to study catalytically essential residues. Further studies by other tools are required to solve these issues.

## Methods

### Generation of Csk mutants

Glutathione S transferase (GST)-Csk fusion proteins were generated and purified as previously described [29]. Csk point mutants were generated using QuikChange (Stratagene) in the parental plasmid and were confirmed by DNA sequencing. Kinase-defective Src (kdSrc) was produced as described previously [23,24].

### Enzyme purification

Bacteria harbouring appropriate plasmids were cultured in LB medium at 37°C with shaking at 250 rpm overnight. The overnight culture was then mixed with an equal volume of fresh LB medium, cooled down to about 20°C. IPTG (0.2 mM) was added to the culture to induce recombinant protein expression at 20°C for 12 hours. The GST fusion proteins were purified by glutathione affinity chromatography as previously described [29]. The purified enzymes were desalted on a Sephadex G25 column equilibrated with the storage buffer (100 mM Tris-Cl, pH 8.0, and 0.1% β-mercaptoethanol). Glycerol was added to the purified fractions to 30% and the enzymes were stored at -20°C. Protein concentration was determined by the Bradford assay and the purity of purified proteins was assessed by SDS-PAGE with coomassie blue staining.

### Kinase activity assay

For assaying PTK activity, phosphorylation of polyE_4_Y and kdSrc was measured using the acid precipitation assay as previously described [7]. Standard kinase assay buffer contains 100 mM EPPS, pH 8, 10% glycerol, 0.1% triton X-100 and 0.1% β-mercaptoethanol. Reaction time for the assays was 10 min. Standard assays used polyE_4_Y at 1 mg ml^-1^, or kdSrc at 10 μM as the phosphate-accepting substrate and ATP at 0.2 mM as the phosphate-donating substrate. To determine the kinase activity at different pH, the kinase buffer contained all the standard buffer components except EPPS was replaced by 100 mM MES or Tris at designated pH. When K_m _and k_cat _were determined with regard to one substrate, the kinase activity was determined at various concentrations of that substrate in the range of 20 to 200 μg ml^-1 ^for polyE_4_Y, 1 to 10 μM for kdSrc or 20 to 200 μM for ATP. When the phosphate-accepting substrate (either polyE_4_Y or kdSrc) was the variable substrate, ATP concentration was 0.2 mM. PolyE_4_Y at 1 mg ml^-1 ^was used when ATP was the variable substrate. The k_cat _and K_m _values were determined by Lineweaver-Burk plots with linear regression using Microsoft Excel. All steady state kinetic assays were performed in duplicate, and repeated at least once. Standard errors were calculated if an assay was performed at least three times.

## Abbreviation used

AC_50_, the concentration of a divalent metal cation that activates Csk to 50% of its full activity; Csk, C-terminal Src kinase; IRK, insulin receptor kinase; PKA, the camp-dependent protein kinase; PTK, protein tyrosine kinase(s); SFK, Src family kinase(s).

## Authors' contributions

XL designed and performed the experiments and analyzed the results. MKA designed and performed experiments. GS designed the experiments, analyzed the results and wrote the paper.
